# Medication tendencies for inducing severe ocular surface symptoms in Japanese Stevens-Johnson Syndrome / toxic epidermal necrolysis patients

**DOI:** 10.1186/2045-7022-4-S3-P88

**Published:** 2014-07-18

**Authors:** Yoshiro Saito, Mayumi Ueta, Ryosuke Nakamura, Emiko Sugiyama, Keiko Maekawa, Yukitoshi Takahashi, Hirokazu Furuya, Akiko Yagami, Setsuko Matsukura, Zenro Ikezawa, Kayoko Matsunaga, Chie Sotozono, Michiko Aihara, Shigeru Kinoshita, Nahoko Kaniwa

**Affiliations:** 1 National Institute of Health Sciences, Division of Medicinal Safety Science, Japan; 2 Kyoto Prefectural University of Medicine, Department of Ophthamology, Japan; 3 Shizuoka Institute of Epilepsy and Neurological Disorders, Division of Clinical Research, Japan; 4 Kochi University Medical School, Department of Aging Science and Neurology, Japan; 5 Fujita Health University, School of Medicine, Department of Dermatology, Japan; 6 Yokohama City University, Graduate School of Medicine, Department of Environmental Immuno-Dermatology, Japan; 7 International University of Health snd Welfare, Atami Hospital, Japan

## Background

Stevens-Johnson syndrome (SJS) and toxic epidermal necrolysis (TEN), are severe cutaneous adverse drug reactions that often induces the mucosal tissue impairments. These symptoms in ocular surface sometimes suffer the patients with pseudomembrane formation leading to vision loss as a sequela. The purpose of this study to reveal the medication tendencies for inducing severe ocular surface complications in SJS/TEN of Japanese populations.

## Method

Japanese SJS/TEN patients were recruited through the participating university/hospitals and nation-wide collecting network (as JSAR research group) from June 2006 to June 2013. Informations including patients' backgrounds, disease symptoms, and administered drugs before the onset of SJS/TEN were also collected. Ocular symptoms were graded as follows; 0; no complication, 1; only hyperemia of the bulbar and palpebral conjunctiva, 2; pseudomembrane formation, 3; defect of the conjunctiva or corneal epithelia. The Grade 2 or 3 was grouped as severe ocular surface complications.

## Results

A total of 197 SJS/TEN patients were recruited; 97 males and 100 females, average age 56.6} 22.3, 23, 115 and 59 for probable SJS, definite SJS and TEN, respectively, and 40, 95, 14 and 48 for eye symptom grade 0, 1, 2 and 3, respectively. Females or young (below 60 years old) patients show higher tendency for developing severe ocular symptoms but these comparisons did not reach the statistical significance. In analysis on drugs or drug groups that more than 14 patients were administered, the SJS/TEN patients with acetaminophen showed significantly higher rate (55.6%) of severe ocular symptoms than those without this drug (28.0%) (p<0.01). The patients with loxoprofen or cephalosporins exhibited the same tendencies with borderline significance. In contrast, no differences in the rates were observed for SJS/TEN related to other drugs such as carbamazepine, allopurinol, quinolones and aspirin. The patients receiving NSAIDs for treatment of cold developed severe ocular symptoms at the rate of 65.4%, while that in the patients administered NSAIDs for other diseases was 17.1%.

## Conclusion

In the Japanese population, we found that acetaminophen or NSAIDs for treatment of cold showed high rates for developing severe ocular complications. In addition to skin, the SJS/TEN patients with these drugs should be intensively treated for eye to prevent severe sequelae.

